# Supplementary figures and images for: An adenosine derivative prevents the alterations observed in metabolic syndrome in a rat model induced by a rich high-fat diet and sucrose supplementation
Source: PLoS One. 2023 Oct 5;18(10):e0292448. doi: 10.1371/journal.pone.0292448 (PMC10553329; doi:10.1371/journal.pone.0292448)

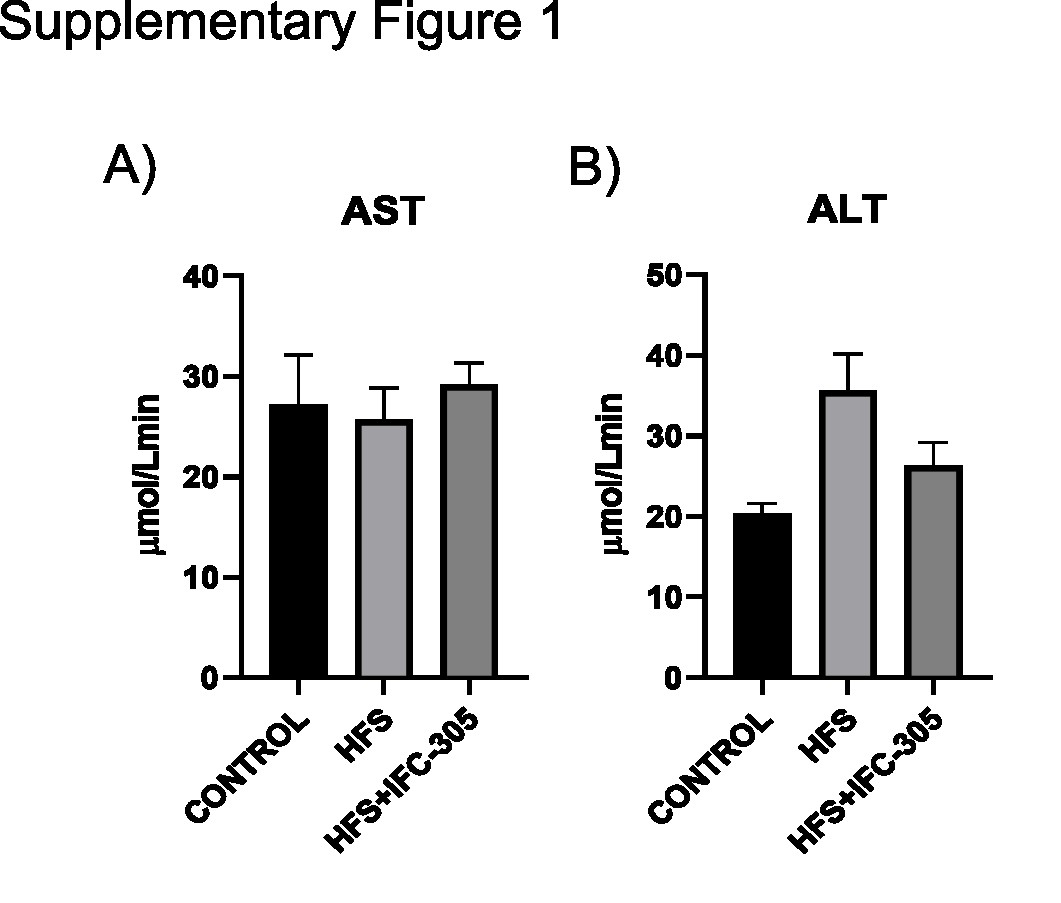

Supplement: S1 Fig — (A) AST and (B) ALT activity was determined in serum from rats in the control (n = 3), HFS (n = 10), and HFS+IFC-305 (n = 10) groups. The values represent the mean of experiments performed in duplicate assays ± SEM. (TIFF) [file pone.0292448.s001.tiff]

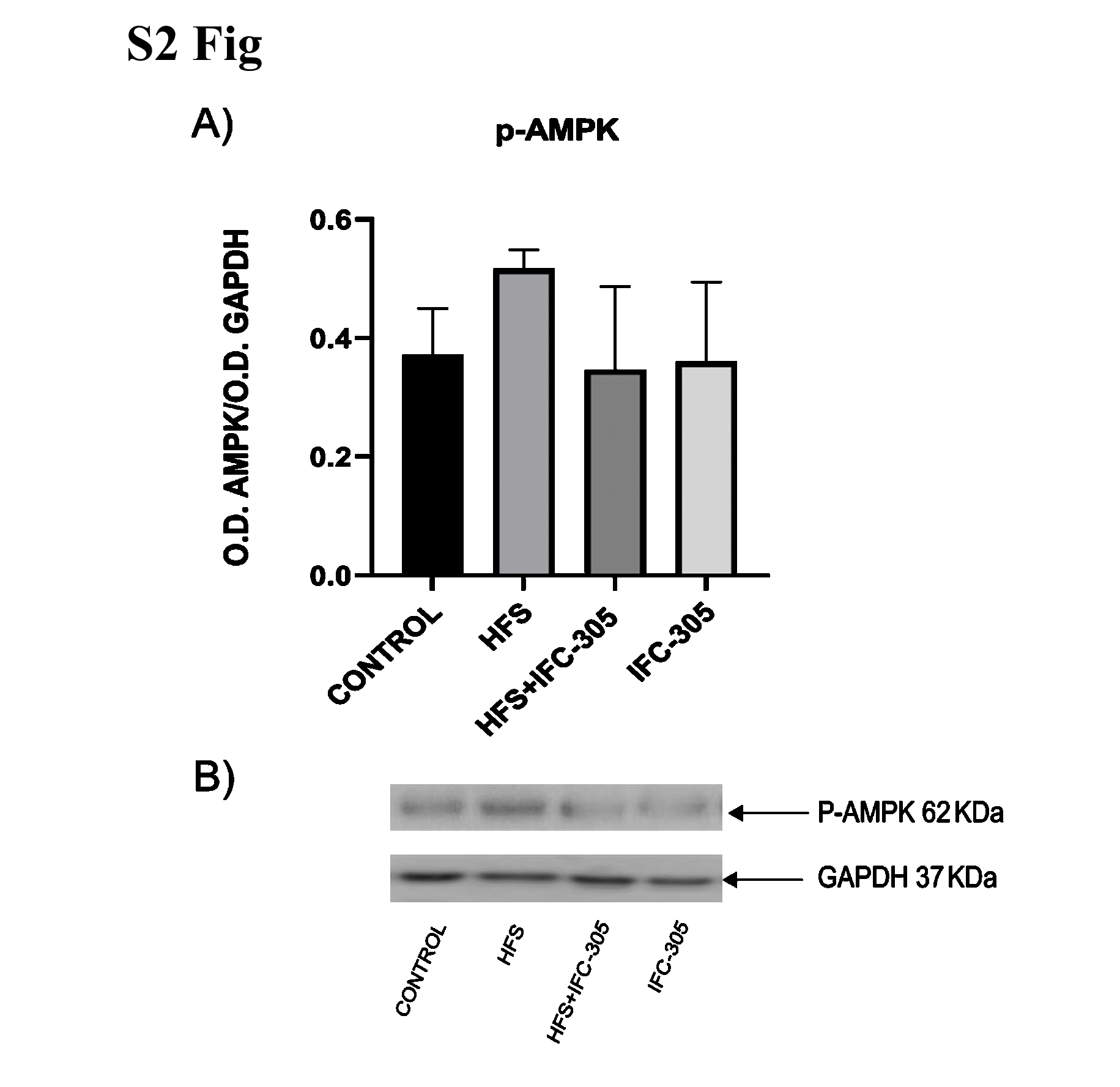

Supplement: S2 Fig — pAMPK protein levels in samples of liver tissue determined by Western blot analysis from rats in the control (n = 3), HFS (n = 4), HFS+IFC-305 (n = 4), and IFC-305 (n = 4) groups. GAPDH was used as an internal control. A) The signal intensities were determined by densitometric analysis of treated blots, and the values were calculated as the ratio of p-AMPK to GAPDH. Each bar represents the mean value of experiments ± SEM. B) Representative blot for each group. (TIFF) [file pone.0292448.s002.tiff]

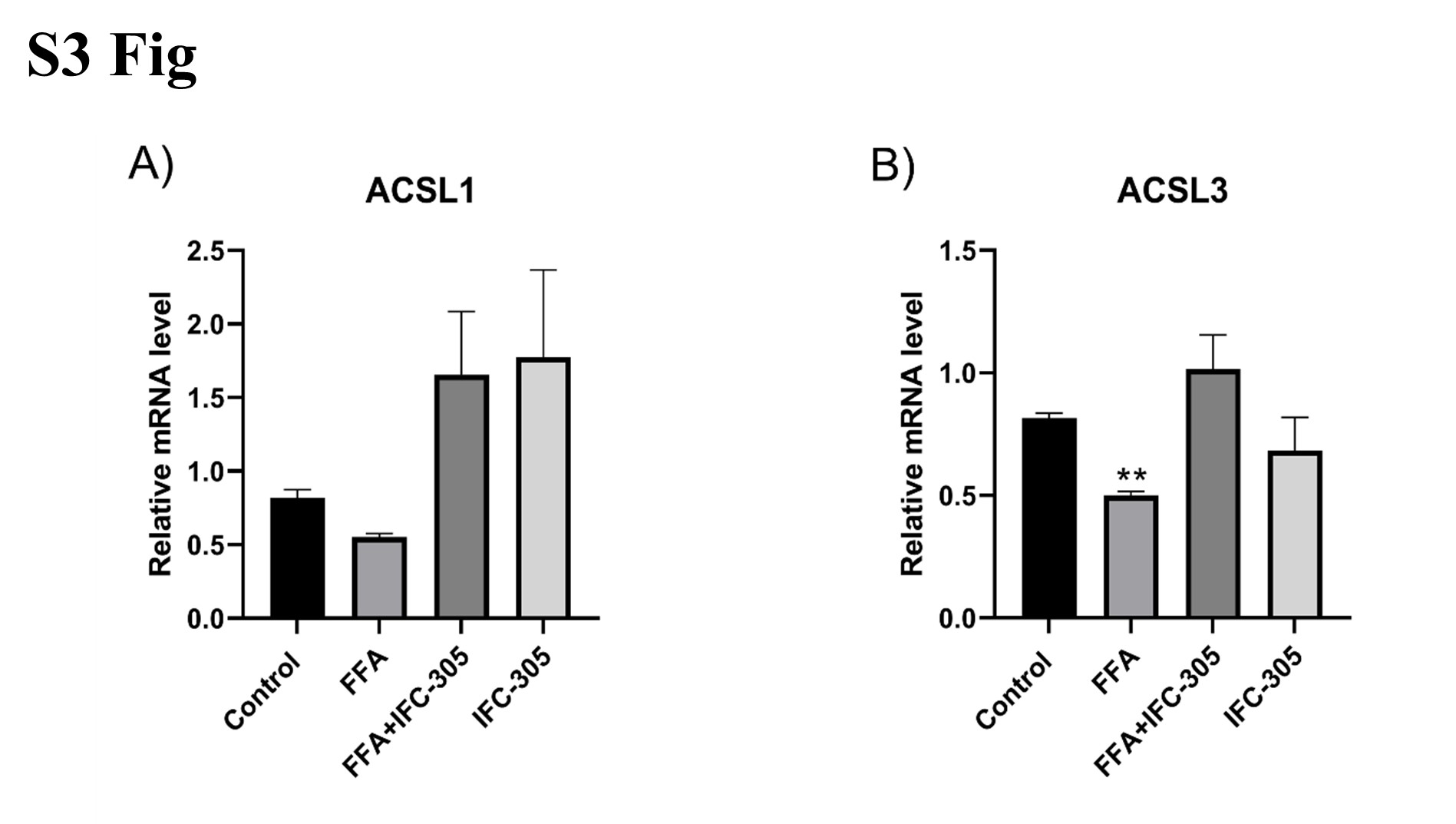

Supplement: S3 Fig — mRNA expression of ACSL1 and ACSL3 in HepG2 cells. HepG2 cells were cultured in serum-free DMEM supplemented with 1% BSA-free FFAs (control) in the presence of 1 mM IFC-305 or FFA with or without 1 mM IFC-305 for 24 h. Real-time quantitative polymerase chain reaction (qPCR) analysis of the mRNA expression of (A) ACSL1 and (B) ACSL3 isoforms normalized to β-actin. The relative mRNA levels were calculated using the comparative ΔΔCt method. mRNA expression is expressed as the mean value ± SEM from three independent experiments. **P < 0.001. (TIFF) [file pone.0292448.s003.tiff]
