# Supplementary material for: An adenosine derivative prevents the alterations observed in metabolic syndrome in a rat model induced by a rich high-fat diet and sucrose supplementation
Source: PLoS One. 2023 Oct 5;18(10):e0292448. doi: 10.1371/journal.pone.0292448 (PMC10553329; doi:10.1371/journal.pone.0292448)
Supplement: S1 Table — Food and water intake (or water+sucrose solution for HFS and HFS+IFC groups) were measured twice a week. Captured data are expressed as mean values ± SEM from 0, 8, 12 and 18 weeks of treatment. Statistical differences were determined with ANOVA followed by a multiple comparison test where “a” indicates a significant difference compared to the control group, P≤0.05. (DOCX) [file pone.0292448.s004.docx]

| **Week** | **Parameter** | **Control** | **HFS** | **HFS+IFC-305** | **IFC-305** |
| --- | --- | --- | --- | --- | --- |
| **0** | Food consumption (g/day) | 25.72±1.45 | 19.14±1.18 ^a^ | 18.98±0.74 ^a^ | 23.31±0.26 |
|  | Water consumption (mL/day) | 25.00±2.27 | 72.65±12.36 ^a^ | 106.74±16.74 ^a^ | 24.35±2.24 |
| **4** | Food consumption (g/day) | 30.97±1.23 | 15.80±0.91 ^a,d^ | 15.57±0.78 ^a^ | 33.60±1.54 |
|  | Water consumption (mL/day) | 32.79±2.38 | 80.36±8.53 ^a^ | 62.90±2.54 ^a^ | 37.99±3.58 |
| **8** | Food consumption (g/day) | 20.40±0.85 | 17.28±1.29 | 16.46±1.27 | 20.37±1.41 |
|  | Water consumption (mL/day) | 30.19±3.58 | 68.99±6.38^a^ | 58.85±9.67 | 32.14±2.08 |
| **12** | Food consumption (g/day) | 22.67±1.22 | 16.40±1.19 ^a^ | 14.13±1.14 ^a^ | 20.77±0.79 |
|  | Water consumption (mL/day) | 32.47±2.67 | 72.24±4.80^a^ | 79.55±6.41^a^ | 32.47±2.46 |
| **18** | Food consumption (g/day) | 23.50±1.5 | 15.38±1.45 ^a^ | 14.19±0.39 ^d^ | 20.00±0.46 |
|  | Water consumption (mL/day) | 35.06±6.99 | 81.58±10.28^a^ | 75.49±4.72^a^ | 31.17±4.12 |

**S1 Tab. Food and water consumption**

Food and water intake (or water+sucrose solution for HFS and HFS+IFC groups) were measured twice a week. Captured data are expressed as mean values ± SEM from 0, 8, 12 and 18 weeks of treatment. Statistical differences were determined with ANOVA followed by a multiple comparison test where “a” indicates a significant difference compared to the control group, p≤0.05.
