# Supplementary material for: An adenosine derivative prevents the alterations observed in metabolic syndrome in a rat model induced by a rich high-fat diet and sucrose supplementation
Source: PLoS One. 2023 Oct 5;18(10):e0292448. doi: 10.1371/journal.pone.0292448 (PMC10553329; doi:10.1371/journal.pone.0292448)
Supplement: Attachment — Submitted filename: Respnse to Reviewers.docx [file pone.0292448.s007.docx]

**Reviewer #1: In general, the authors revised the manuscript appropriately based on the reviewers’ comments. However, several minor mistakes are remaining.**

**1. Fig. 3: “INF-γ” should be revised as “IFN-γ.”**

Answer: In Fig. 3 “INF-γ” was changed by “IFN-γ.”

**2. “N”, “L”, and “GA” are not used in Fig. 4 but explained in the legend of Fig. 4. The authors should either include them in the figure or exclude them form the legend.**

Answer: “N”, “L”, and “GA” were removed from the legend of Fig. 4

**Reviewer #2: The authors answered all my questions adequately.
In the future, I would like the authors to use the F4/80 analysis because it is very useful for analyzing inflammation.**

Thank you very much for the recommendation, we agree that the F4/80 antibody would be very useful to evaluate inflammation.
